# Case Report: Giant Biatrial Myxoma Mimicking Malignant Cardiac Tumor in a Patient With a Hepatic Angiomatous Mass

**DOI:** 10.3389/fcvm.2021.676807

**Published:** 2021-05-28

**Authors:** Chengming Fan, Hao Zhang, Huanwei Zhuang, Zenan Jiang, Haoyu Tan, Chukwuemeka Daniel Iroegbu, Long Song, Liming Liu

**Affiliations:** ^1^Department of Cardiovascular Surgery, The Second Xiangya Hospital, Central South University, Changsha, China; ^2^Department of Cardiothoracic Surgery, Central South University Xiangya School of Medicine Affiliated Haikou Hospital, Haikou, China

**Keywords:** giant biatrial myxoma, malignant, surgery, atrial fibrillation, Cox-Maze IV procedure

## Abstract

Cardiac myxomas, primarily originating from the left atrium, are the most prevalent types of benign cardiac tumors; however, biatrial myxomas are extremely rare. Herein, we present a rare case of a 55-year old male with exertional dyspnea and intermittent chest discomfort due to a giant biatrial mass with concomitant atrial fibrillation and hepatic hemangioma. The giant tumor with its peduncle at the interatrial septum involved both atria; however, bulging through the tricuspid valve to the right ventricle during systole. Hence, excision of the giant cardiac tumor (which grossly composed of three parts: stiff, fleshy, and soft) and Cox-Maze IV procedure was performed with the resected specimen measuring 100 × 80 × 40 mm. The patient who was in a stable condition was discharged home on the 12th post-operative day. Thus, given the excellent post-operative results achieved, surgical treatment in large multi-cavitary benign cardiac tumors is feasible and should be considered a potentially curative therapy.

## Background

Cardiac myxoma, mostly found within the left atrium, is the most prevalent primary cardiac tumor in adults (1). Cardiac myxomas accounts for 30–50% of all primary tumors of the heart with an annual incidence of 0.5 per million populations (2, 3). The majority (60–88%) occurin the left atrium, with a smaller proportion in the right atrium (4–28%) and in rare cases in left ventricular (8%), right ventricular (2.5–6.1%), biatrial (<2.5%), or multiple locations (2.5%) (4, 5). Myxomas developing in three to four heart chambers are extremely rare and usually considered malignant cardiac tumors, especially when tumors located in other organs were detected (6–8). We report a case of a biatrial tumor, which occupies both atria and right ventricle with concomitant atrial fibrillation and hepatic hemangioma.

## Case Presentation

A 55-year-old man with a 2-year history of shortness of breath after slight activity with over 2 weeks of severe chest pain (without any anginal characteristics) was referred to our center. Occasional dizziness with no headaches, fever, or cough was dictated with a body temperature of 36.1°C and blood pressure of 135/111 mmHg at resting conditions. Physical examination revealed that the pulse was 78 bpm, while the heart rate was 97b pm with an irregular heart rhythm. The first heart sound (S1) intensity varied, and a regurgitant murmur was heard at the apex. There were no other notable clinical findings and family history of cardiovascular disease following medical history and physical examination. Laboratory tests revealed that the N-terminal brain natriuretic peptide precursor (NT-ProBNP) was increased (1421.0 pg/ml), while CK, CK-MB, and cTnT were normal.

**Figure 1:**
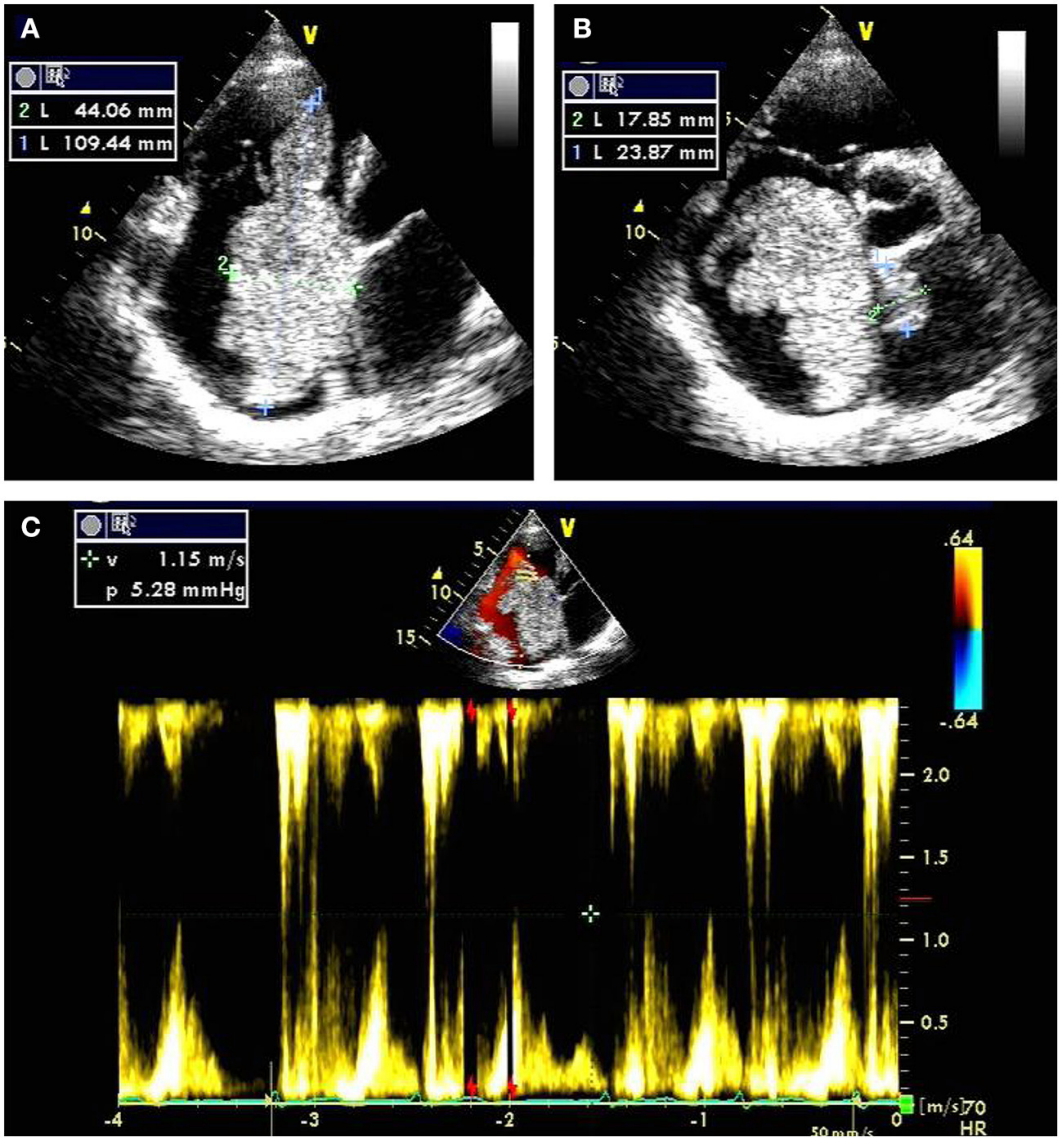
Transthoracic echocardiography preoperatively showing a giant biatrial mass with the size of 44.06 mm × 109.44 mm in the right atrium **(A)** and 17.85 mm × 23.87 mm in the left atrium **(B)**; Color Doppler flow imaging showing severe mechanical hemodynamic obstacles in the right atrium primarily occupied by the giant tumor tissue **(C)**.

Electrocardiography revealed atrial fibrillation with inverted T-waves. Transthoracic echocardiography demonstrated a giant biatrial mass with 44.06 mm × 109.44 mm in the right atrium (Figure 1A) and 17.85 mm × 23.87 mm in the left atrium (Figure 1B). Notably, the blood flowed through the tumor tissue's space with severe mechanical hemodynamic obstacles (Figure 1C). The diameters of RV, LA, and RA were 46, 43, and 57, respectively. The left ventricular systolic function was impaired with an ejection fraction of 42%. Mild mitral and tricuspid regurgitation were also detected.

**Figure 2:**
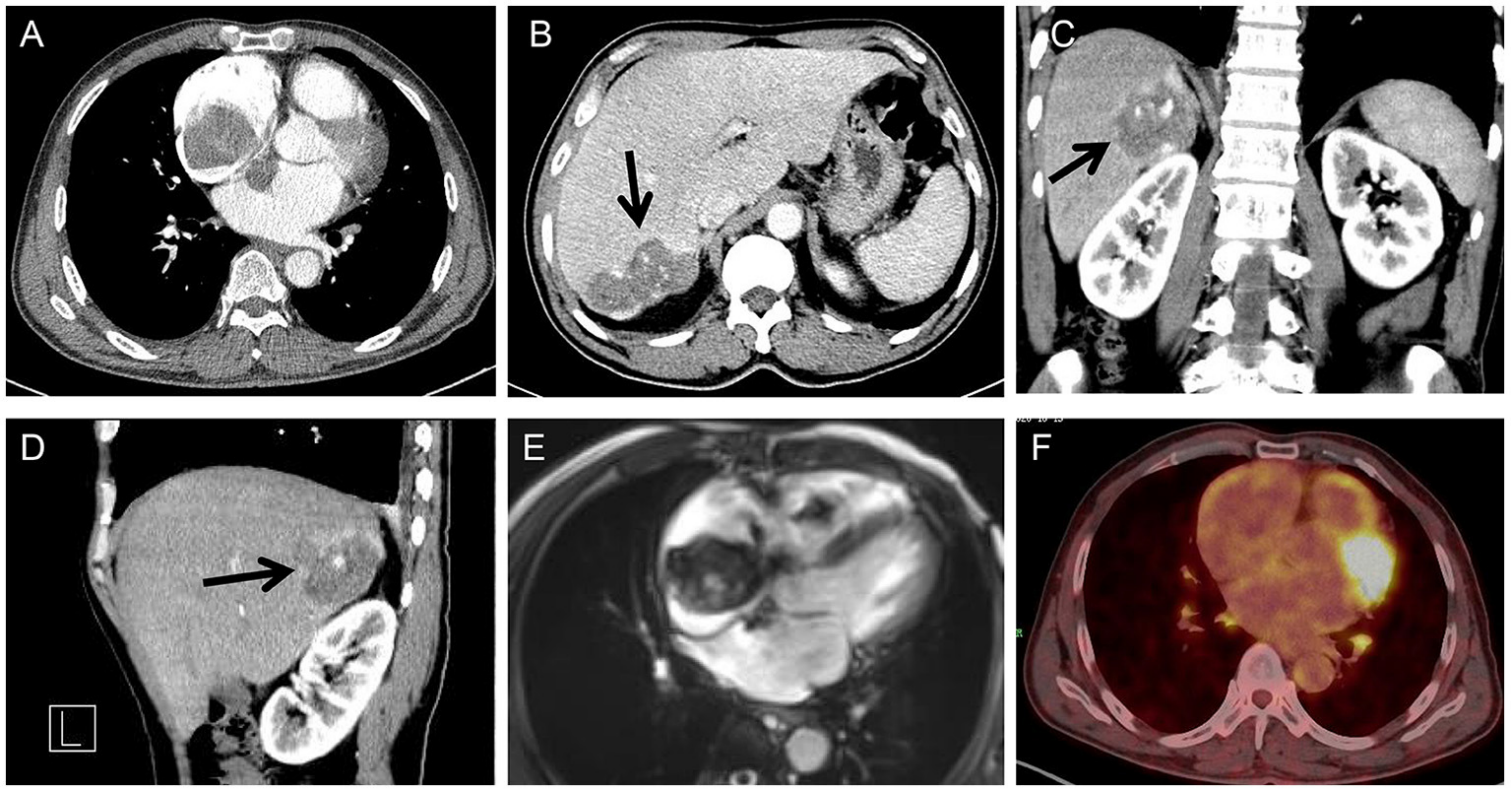
Cardiac CT showing a biatrial mass with the atrial septum infiltrated **(A)**; Abdominal CT showing an angiomatous mass in the right posterior lobe of the liver (arrow) (transverse view) **(B)**, coronal **(C)**, and sagittal **(D)** plane; cardiac MRI showing a giant biatrial mass with tricuspid valve involved **(E)**; PET-CT showing a giant mass with increased glucose metabolism in the right atrium **(F)**.

Thoracic and abdominal computer tomography (CT) showed a biatrial mass (Figure 2A) and an angiomatous mass in the right posterior lobe of the liver (Figures 2B–D, arrows). Cardiac magnetic resonance imaging (MRI) was also performed, which showed a giant biatrial mass involving the tricuspid valve and the atrial septum. The tumor was unevenly enhanced on a contrast scan, suggesting a high possibility of a malignant tumor (Figure 2E). A Positron Emission Tomography-CT (PET-CT) scan was also performed, which detected an FDG-avid lesion in the right atrium. No other FDG-avid lesions are demonstrated. The possibility of a malignant tumor of mesenchymal origin was considered, given the MRI and CT scan results combined with an enhanced PET-CT scan (a giant soft tissue-like mass with increased glucose metabolism in the right atrium) (Figure 2F).

Likewise, the possibility of a malignant tumor of mesenchymal origin was considered, given the blade-like low-density foci in the right liver as shown in the enhanced CT scan. A slightly enlarged lymph node with a slight increase in glucose metabolism in the left axilla and bilateral hilar was considered reactive lymph node hyperplasia. After extensive discussions with the patient and his family, excision of the giant cardiac tumor, tricuspid valvuloplasty, and Cox-Maze-IV procedure was scheduled.

**Figure 3:**
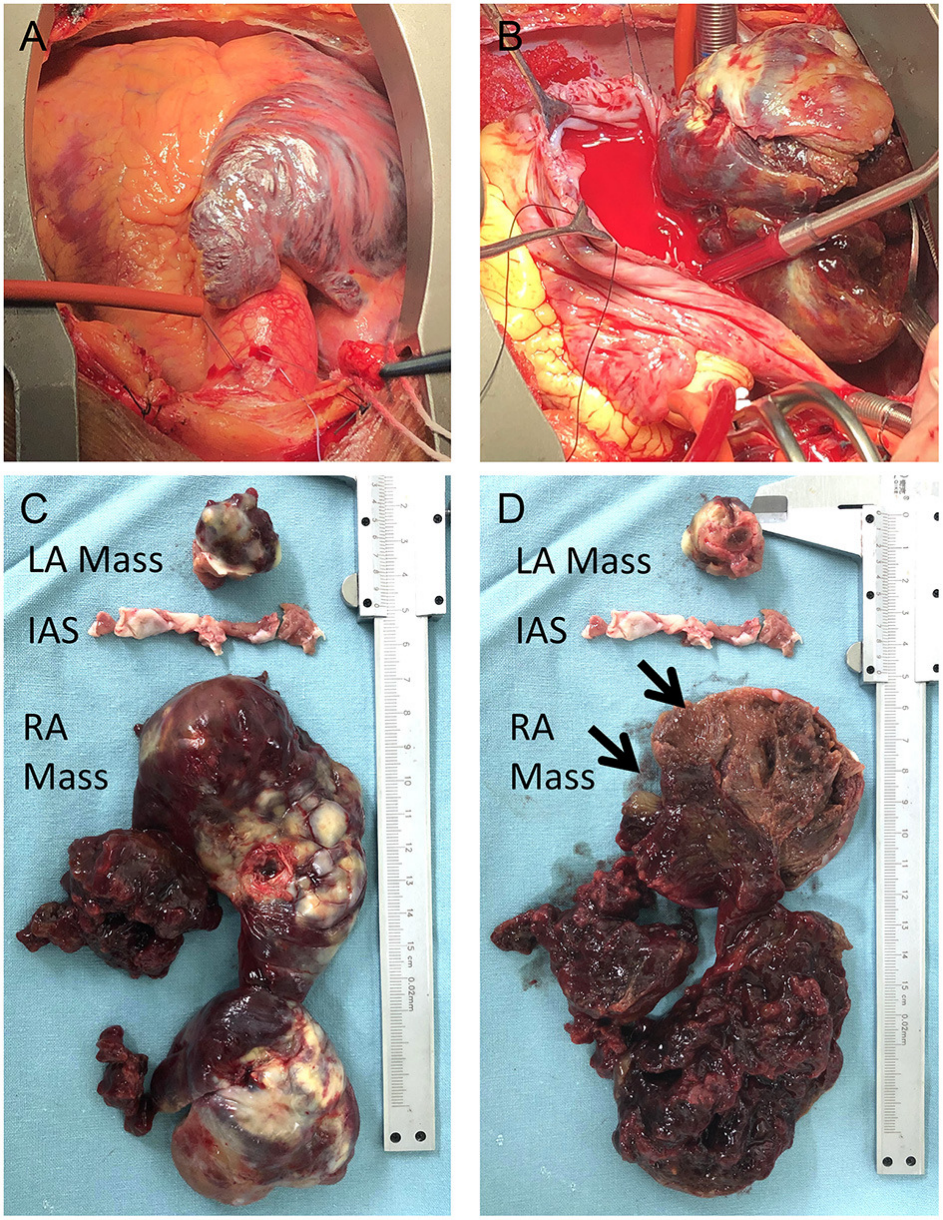
Intraoperative view of the tumor: right atrium was enlarged **(A)** with the tumor inside the chamber; The right side of the tumor was visualized with the opening of the right atrium **(B)**; the biatrial mass together with the infiltrated septum was resected **(C,D)**; the right atrial lesion was grossly composed of two parts (**D**, arrows).

A standard median sternotomy incision was performed, and on opening the pericardium, a massive right atrium was visualized (Figure 3A). The aorta was then cannulated. Separate cannulas were placed in the superior vena cava (SVC) and inferior vena cava (IVC). After full heparinization, cardiopulmonary bypass (CPB) was routinely applied. The aorta was cross-clamped, and a cold cardioplegic solution (Del Nido) was instilled via the aortic root to arrest the heart. Following the right atrium's opening, a careful exploration of the abnormalities was completed (Figure 3B). Together with the involved septum, the biatrial tumor was removed (Figures 3C,D). The resected mass from the right atrium was grossly composed of two parts; a cutaneous soft tissue-like mass and a peanut-shell shape-like mass (Figure 3D, arrows).

Radiofrequency ablation with Cox-Maze-IV procedure, reconstructing the atrial septum with a suitable bovine pericardial patch, and tricuspid valvuloplasty with the implantation of a prosthetic ring (size 32, Sorin sovering band) were sequentially performed. After careful hemostasis and closing of the wound in layers, the patient was carefully transferred to the intensive care unit (ICU) in a stable condition. Early post-operative management, including continuous arterial blood pressure monitoring and ventilation, was created to stabilize circulation. Transthoracic echocardiography and electrocardiogram were performed to capture any hint of residual tumor or arrhythmias, especially A-V block.

**Figure 4:**
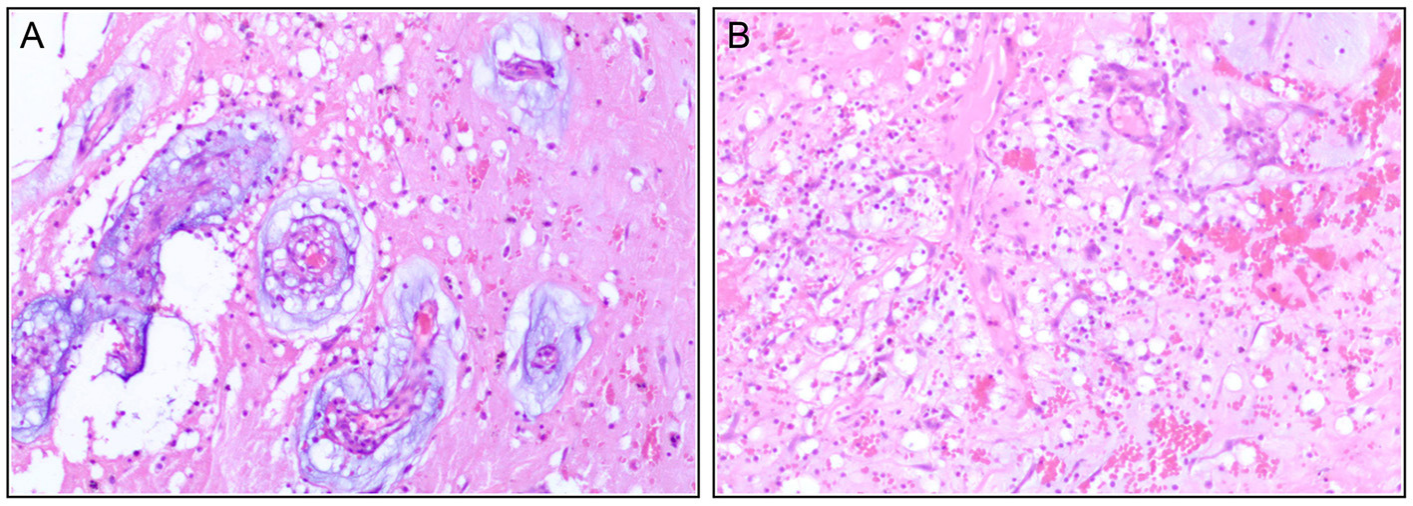
Immunohistochemistry post-operatively confirm the diagnosis of the cardiac myxoma with interstitial fibrous hyperplasia **(A)**, hemorrhage **(B)**, necrosis, and plasmacytes infiltrate.

Pathology results confirmed the primary diagnosis of cardiac myxoma and showed interstitial fibrous hyperplasia with vitrification and calcification, hemorrhage, necrosis, and plasmacytes infiltrates (Figures 4A,B). The patient recovered with sinus rhythm without further complications and was discharged on the eighth post-operative day in a stable condition with recommended subsequent follow-up.

## Discussion and Conclusions

Cardiac myxoma is the most prevalent primary tumor within the heart cavity. It is believed to be benign, with some complex manifestations with malignant tendencies (8, 9). Typical myxomas are isolated, single, smooth surface, spherical, pedicled, variable in size (~1–8 cm in diameter, mean 5 cm), 90% located in the left atrium, attached to the atrial septal fossa ovale, and extending into the affected chamber of the heart (1). In extreme cases, the cavity can be filled by the tumor mass (6). The tumor may prolapse toward the ventricle during diastole, as shown in the present case. However, because symptoms may mimic other cardiac conditions, the preoperative diagnosis of myxoma becomes difficult (10, 11). Besides, giant biatrial myxomas involving the right ventricular cavity are extremely rare (12).

A thrombus, liquefied viscose tissue, and a liquefied viscose mesenchymal tumor, including the heart's lymphoma, are the most common differential diagnosis of cardiac myxoma (13). Myxoma looks similar to thrombosis, especially a globular thrombosis in the atrium. However, myxoma and thrombosis are two different kinds of pathology. Significantly, the thrombus's surface is not covered with cells, and its mechanization process starts from within its wall. On the contrary, the non-degenerative parts and the myxoma proximal pedicle are covered with cells, while tissue necrosis and fibrinoid degeneration are in the distal part. Notably, the myxomatoid mesenchymal tumor of the heart is most easily mixed with myxoma. In the presented case, the mesenchymal origin was indicated mainly based on the location (left and right atrium), the blade-like low-density foci in the right liver as shown in the enhanced CT scan. However, other histologies should also be considered. The final result from the pathology would confirmed the diagnosis. Thus, adequate histologic sections, comprehensive observation, and cell identification are crucial for accurate diagnosis (13–15).

Interestingly, studies show that cardiac myxoma may present with possible malignant degeneration and malignant clinical behavior (8, 9, 16, 17). Besides, cardiac myxoma can be preoperatively misdiagnosed as malignant tumors, especially when extra-cardiac tumor-like anomalies are detected (14). Hepatic malignant mass was recently reported to coexist with cardiac myxoma (18). However, the final diagnosis of the angiomatous liver mass in the reported case was unknown. Unfortunately, given the patient's financial status and the lack of basic health insurance, a further clinical investigation was not carried out, which would have identified the asymptomatic angiomatous liver mass. Myxomas in males are very rare but can be seen in the setting of Carney Complex, an autosomal disorder due to mutation of the PRKA R1A subunit of PKA. Patients with these mutations have other features like spotty skin pigmentation, endocrine tumors, peripheral nerve tumors, and a familial predisposition (19). In the presented case, the patient had no adrenal tumors or skin freckles. He denied any previous medical or family history. It is believed that patients with cardiac tumors, both malignant and benign tumors, should undergo surgery in a timely fashion in a specialized center (6). If the myxoma is huge, the blood can only flow through the tumor tissue's space, leading to severely unstable hemodynamics due to mechanical obstacles.

It is critically essential to note that cardiac myxoma tissues are loose and fragile and easy to break off from the tumor (20). Thus, surgical resection should be considered once cardiac myxoma is diagnosed. Notably, the prognosis after surgery is usually excellent in the case of benign tumors (21), even when the involved myxoma is multi-cavitary (4, 22).

It is worth noting that, before the aorta is occluded during the operation, intracardiac and extracardiac exploration should be avoided. The atrial septum or atrial wall, endocardia, and myocardium at the tumor pedicle should be removed entirely. If the heart valves are invaded and cannot be repaired, then valvular replacement should be performed. Valvular annuloplasty should be performed if annular insufficiency from the enlargement was observed. Also, a patch repair should be performed if an extensive range of atrial septal resection could not be avoided. After tumor removal, the heart cavity should be flushed thoroughly to prevent tumoral debris from remaining in the heart chamber. Also, surgery must include resection of all abnormal tissues (23).

In the case herein, the patient had suspected malignant cardiac tumor and other cardiac lesions, including atrial fibrillation, valvular stenosis, or insufficiency. Thus, total resection with radiofrequency ablation and valvuloplasty for the giant tumor was performed to maximize the patient's benefit. Significantly, follow-up with echocardiography should be continued to detect recurrence and long-term effect.

## Data Availability Statement

The original contributions presented in the study are included in the article/supplementary material, further inquiries can be directed to the corresponding author/s.

## Ethics Statement

The studies involving human participants were reviewed and approved by the study protocol was approved by the Ethics Committee of the Second Xiangya Hospital of Central South University, Changsha, China. The patients/participants provided their written informed consent to participate in this study. Written informed consent was obtained from the individual(s) for the publication of any potentially identifiable images or data included in this article.

## Author Contributions

CF drafted the manuscript. CF and LL designed the study. HZha, CI, ZJ, and LS revised the manuscript. ZJ, HT, and HZhu were responsible for the collection of data or analysis. All authors read and approved the final manuscript.

## Conflict of Interest

The authors declare that the research was conducted in the absence of any commercial or financial relationships that could be construed as a potential conflict of interest.
